# Pathological complete response and long-term outcomes after neoadjuvant therapy for breast cancer: a multicenter retrospective cohort study with exploratory subtype analyses

**DOI:** 10.3389/fonc.2026.1994688

**Published:** 2026-10-23

**Authors:** Shaolong Fu, Kaiyang Xiao, Sibao Huang, Zhishan Chen, Debo Chen, Weibin Lian

**Affiliations:** 1 Department of Thyroid and Breast Surgery, Nan’an Hospital, Quanzhou, Fujian, China; 2 Department of Breast Surgery, Quanzhou First Hospital Affiliated to Fujian Medical University, Quanzhou, Fujian, China

**Keywords:** breast cancer, breast cancer subtype, neoadjuvant therapy, pathological complete response, survival

## Abstract

**Background:**

To examine differences in pathological complete response (pCR) rates across immunohistochemistry (IHC)-defined breast cancer surrogate subtypes and the association of pCR with recurrence-free survival (RFS) and breast cancer-specific survival (BCSS).

**Methods:**

This retrospective multicenter study included 3,803 patients from the Shanghai Jiao Tong University Breast Cancer Database who received neoadjuvant therapy followed by surgery. Total pCR was defined as ypT0/is ypN0. Multivariable logistic and Cox regression assessed factors associated with pCR and its association with survival, respectively. Exploratory global subtype-by-pCR interaction tests were restricted to the remaining four subtypes with estimable within-subtype pCR contrasts. Sensitivity analyses included exploratory covariate-balancing analyses based on propensity score matching and overlap weighting, calendar-year adjustment, and subtype-stratified Cox models.

**Results:**

Overall, 908 patients achieved pCR (23.88%), with rates differing across IHC-defined surrogate subtypes (P < 0.001). During a median follow-up of 46 months, pCR was associated with favorable RFS (adjusted HR 0.37, 95% CI 0.24–0.57) and BCSS (adjusted HR 0.30, 95% CI 0.18–0.50; both P < 0.001). Exploratory global interaction tests among the remaining four subtypes with estimable within-subtype pCR contrasts showed no statistical evidence of heterogeneity for RFS (P for interaction = 0.665) or BCSS (P for interaction = 0.718); Luminal A-like disease could not be included in these tests because no RFS or BCSS events occurred among the five patients who achieved pCR. Exploratory propensity-score matching yielded 907 pairs; matched-pair robust HRs were 0.43 (95% CI 0.27–0.69) for RFS and 0.38 (95% CI 0.22–0.64) for BCSS. Associations were consistent across the other sensitivity analyses.

**Conclusion:**

pCR frequency varied across IHC-defined surrogate subtypes, and pCR was associated with favorable survival in the overall cohort. The exploratory interaction analyses did not establish effect modification among the four included subtypes; Luminal A-like disease could not be assessed for interaction because of sparse events. Subtype-specific survival estimates remain exploratory and require confirmation in adequately powered studies.

## Introduction

Breast cancer remains the most frequently diagnosed malignancy and the leading cause of cancer-related death among women worldwide, accounting for a substantial global health burden (1). With improvements in systemic therapy and earlier detection, treatment paradigms for early and locally advanced breast cancer have evolved considerably over the past decade (2). Neoadjuvant therapy, initially reserved for inoperable or locally advanced disease, has now become a standard treatment strategy for many patients with stage II–III breast cancer, particularly those with triple-negative breast cancer (TNBC) and human epidermal growth factor receptor 2 (HER2)-positive subtypes (2). Beyond tumor downstaging and increasing breast-conserving surgery rates, neoadjuvant treatment offers a unique *in vivo* platform for assessing therapeutic sensitivity and tailoring postoperative management according to residual disease burden (2).

Pathological complete response (pCR), commonly defined as the absence of residual invasive disease in the breast and axillary lymph nodes after neoadjuvant therapy, has emerged as one of the most important short-term endpoints in breast cancer (3, 4). Multiple studies have demonstrated that patients achieving pCR generally experience superior long-term outcomes, including improved event-free survival and overall survival (3). On this basis, U.S. Food and Drug Administration guidance allows pCR to be considered as a potential endpoint to support accelerated approval in selected patients with high-risk early breast cancer (5). However, the strong association between pCR and prognosis at the individual-patient level should not be interpreted as establishing pCR as a validated trial-level surrogate for long-term survival outcomes. Moreover, whether the magnitude of the prognostic association between pCR and long-term outcomes differs across biological subtypes remains uncertain and should be assessed using formal interaction testing rather than inferred from subgroup-specific estimates alone (3, 4).

The probability of achieving pCR varies across breast cancer subtypes, with higher rates generally observed in HER2-positive and triple-negative disease than in hormone receptor-positive/HER2-negative disease (3). Differences in response frequency should be distinguished from potential differences in the prognostic association of pCR. Previous pooled analyses have examined the latter association across subtypes (4, 6), but whether similar patterns are evident in heterogeneous real-world populations requires further assessment.

Although the prognostic association between pCR and long-term outcome is well established, important uncertainties remain regarding how consistently this association is observed across biologically distinct breast cancer subtypes in routine clinical practice. Previous studies have differed in pCR definitions, survival endpoints, treatment eras, and analytical approaches, while relatively few large multicenter real-world cohorts have simultaneously evaluated determinants of pCR, both recurrence-free survival (RFS) and breast cancer-specific survival (BCSS), and potential subtype-related effect modification using formal interaction testing (3, 4, 6). Therefore, rather than seeking to redefine the established prognostic role of pCR, the present study aimed to extend existing evidence by evaluating the pCR–survival association in a large real-world cohort derived from a multicenter database, quantifying differences in pCR occurrence across IHC-defined surrogate subtypes, and exploring potential subtype-related heterogeneity in this association.

## Materials and methods

### Study design and patient population

This retrospective study used the Shanghai Jiao Tong University Breast Cancer Database (SJTU-BCDB), which integrates records from 42 medical centers across China. The analytic cohort comprised 3,803 patients treated between 2012 and 2021 with histologically confirmed invasive breast cancer who received neoadjuvant therapy followed by surgery and had the clinicopathological, treatment, surgical pathology, pathological response, and endpoint-specific follow-up information required for analysis. Patients with *de novo* metastatic disease or non-invasive breast malignancy were not included. Source-level screening and criterion-specific exclusion counts were not retained and could not be reconstructed. The study followed the Declaration of Helsinki and was approved by the Ethics Committee of Quanzhou First Hospital Affiliated to Fujian Medical University. Data were de-identified, and informed consent was waived because of the retrospective design.

### Variables and definitions

Clinical variables included age, body mass index (BMI), menopausal status, clinical T stage (cT), and clinical N stage (cN). Estrogen receptor (ER) and progesterone receptor (PR) positivity required ≥1% nuclear staining by immunohistochemistry (IHC) (7). HER2 positivity required IHC 3+ or gene amplification; IHC 2+ tumors were classified using confirmatory *in situ* hybridization (ISH)/fluorescence *in situ* hybridization (FISH) (8). Because gene-expression profiling was not available, “subtype” denotes operational IHC-defined clinicopathological surrogate categories rather than intrinsic molecular subtypes (9). Hormone receptor (HR) positivity denotes ER and/or PR positivity. Luminal A-like tumors required ER positivity, HER2 negativity, PR expression ≥20%, and Ki-67 <20% (9). The database algorithm assigned all other HR-positive/HER2-negative tumors to Luminal B-like/HER2-negative, including ER-positive tumors with PR expression <20% or Ki-67 ≥20% and ER-negative/PR-positive tumors. HER2-positive tumors were classified by ER status: ER-positive tumors were Luminal B-like/HER2-positive, and ER-negative tumors were ER-negative/HER2-positive irrespective of PR or Ki-67. TNBC required ER, PR, and HER2 negativity. Ki-67 was recorded as a continuous percentage. The PR and Ki-67 thresholds were operational classification criteria, not universal predictive or prognostic cut-offs. Assay details and central pathology review were not consistently available; inter-laboratory Ki-67 variability therefore cannot be excluded (10, 11).

Neoadjuvant platinum exposure and anti-HER2 therapy were analyzed as binary covariates. Anti-HER2 regimens were additionally described as trastuzumab alone, trastuzumab plus pertuzumab, trastuzumab plus pyrotinib, or pyrotinib alone. Chemotherapy regimen labels are summarized in **Supplementary Table S3**; 1,809 patients (47.6%) were recorded as “Other,” comprising heterogeneous non-standardized descriptions. Regimen backbone, sequencing, dose density, treatment intensity, and treatment-cycle information could not be standardized reliably for adjustment. A standardized indicator of neoadjuvant endocrine therapy was unavailable. Calendar year of surgery was used as a proxy for treatment era. Postoperative chemotherapy, radiotherapy, and endocrine therapy were available as broad indicators. Specific post-neoadjuvant agents, including trastuzumab emtansine (T-DM1) and capecitabine, and perioperative/postoperative immunotherapy use were not identifiable. Standardized information on endocrine agents, treatment duration, modifications, and adherence was unavailable.

Pathological complete response (pCR) was defined as the absence of residual invasive cancer in both the breast and axillary lymph nodes (ypT0/is ypN0). Breast pCR was defined as the absence of residual invasive cancer in the breast (ypT0/is). Unless otherwise specified, pCR refers to total pCR.

### Study endpoints

The primary endpoint was pCR; survival outcomes were RFS and BCSS. RFS was measured from surgery to the first documented local, regional, or distant recurrence or death from any cause. BCSS was measured from surgery using the database-provided breast cancer-specific death indicator (1 = breast cancer-specific death; 0 = otherwise) and corresponding follow-up time; observations without such an event were censored at the supplied time. Separate indicators and times for non-breast cancer deaths were unavailable, and database documentation did not specify whether these deaths were censored at their actual dates; this rule could not be independently verified. Patients without an observed endpoint event were censored at their last database-recorded follow-up. A standardized loss-to-follow-up indicator was unavailable, precluding calculation of a distinct loss-to-follow-up rate. Median follow-up was 46 months (interquartile range, 35–60 months).

### Statistical analysis

Continuous variables were summarized as mean ± standard deviation (SD) and compared using Student’s t-test or the Mann–Whitney U test, guided by Shapiro–Wilk testing. Categorical variables were summarized as n (%) and compared using χ² tests. Overall subtype differences in pCR rates used Pearson’s χ² test, followed, if significant, by two-sample proportion tests without continuity correction and Holm adjustment across all 10 pairwise comparisons.

Univariable and multivariable logistic regression evaluated factors associated with pCR; Kaplan–Meier estimates and log-rank tests described unadjusted survival. Univariable and multivariable Cox models evaluated RFS and BCSS. Primary adjusted models included prespecified covariates: age, BMI, clinical T and N stage, subtype, platinum-based chemotherapy, and anti-HER2 therapy; Cox models additionally included pCR. Estimates were reported as odds ratios (ORs) or hazard ratios (HRs) with 95% confidence intervals (CIs). Primary Cox models used Efron ties and Wald-based CIs and P values. Covariate-specific and global Schoenfeld residual tests assessed proportional hazards; subtype non-proportionality prompted subtype-stratified Cox analyses. Postoperative therapies were described rather than entered as baseline covariates because they followed response assessment and could lie on the post-response pathway; start dates were unavailable for time-dependent modeling. Separate non-breast cancer death indicators and times were unavailable, precluding competing-risk analyses.

Exploratory subtype-specific Cox models included pCR and the same adjustment covariates excluding subtype, using Efron ties. Diagnostics recorded fitted parameter counts excluding baseline hazards, coefficients aliased because of absent within-subtype variation, convergence warnings, and coefficient and standard-error finiteness (**Supplementary Table S6**). In the unmatched, unweighted cohort, 3-df likelihood-ratio tests compared four-subtype models containing pCR and subtype main effects with and without pCR-by-subtype terms, using the same adjustment and Efron ties. Luminal A-like disease was excluded because its five pCR patients had no RFS or BCSS events, causing monotone likelihood in five-subtype models; it remained in primary and descriptive analyses, with its subgroup pCR HR not estimable. Term-specific and global Schoenfeld tests evaluated interaction models. Subtype-stratified models with common versus subtype-specific pCR coefficients were also compared by 3-df likelihood-ratio tests using the same four-subtype cohort, adjustment, and ties (**Supplementary Table S7**).

In the primary logistic and Cox outcome models, ER, PR, HER2, and Ki-67 were not entered separately because they defined subtype. A classification sensitivity analysis merged the HER2-negative luminal groups and classified HER2-positive tumors by combined ER/PR status, yielding HR-positive/HER2-negative, HR-positive/HER2-positive, HR-negative/HER2-positive, and TNBC categories. HR positivity required ER and/or PR positivity, whereas HR negativity required both ER and PR negativity. Logistic regression retained the other primary covariates and used HR-positive/HER2-negative disease as reference (**Supplementary Table S4**). These classification and reference changes precluded direct comparison with five-subtype estimates or isolation of the effects of removing the PR/Ki-67 distinction; Ki-67 measurement variability was not quantified.

Primary model covariates were complete for all 3,803 patients; no covariate-related exclusions or imputation were required. Incomplete or heterogeneous ancillary variables were described (**Supplementary Table S5**). Selection related to source-level missingness could not be assessed because screened-record and criterion-specific exclusion counts were unavailable.

Because pCR is a post-neoadjuvant response state rather than an assigned intervention, propensity-score matching (PSM) and overlap weighting assessed covariate-balanced associations rather than causal effects. Logistic regression estimated pCR propensity scores (PS) using age, BMI, menopausal status, clinical T and N stage, ER status, PR status, continuous Ki-67, subtype, platinum-based chemotherapy, anti-HER2 therapy, and calendar year of surgery. Marker variables captured information not fully represented by subtype, which also incorporated HER2; PS coefficients were not interpreted as independent biological effects. Nearest-neighbor 1:1 matching without replacement used exact subtype matching and a caliper of 0.2 SDs of the logit PS. Cox models used matched-pair clustered robust standard errors; matching targeted the covariate distribution of matchable pCR patients.

Using the same PS, overlap weights were 1 − PS for pCR and PS for non-pCR. Weighted Cox models used robust standard errors. Balance before and after matching or weighting was assessed using absolute standardized mean differences (SMDs), with <0.10 indicating adequate balance. Overlap weighting targeted the population with greatest overlap in estimated pCR probabilities; its HRs and matched HRs were complementary associative estimates. Neither approach addressed unmeasured center or treatment-related factors. Additional logistic and Cox models adjusted for categorical calendar year of surgery. Unavailable center identifiers precluded center-stratified, shared-frailty, or center-clustered models. Analyses used R 4.6.0; two-sided P < 0.05 indicated statistical significance. This study was conducted and reported in accordance with the STROBE guidelines for observational studies (12).

## Results

### Patient characteristics and distribution of pCR

A total of 3,803 patients were included, of whom 908 achieved pCR, corresponding to an overall rate of 23.88% (**Table 1** and **Supplementary Figure S2**). Baseline characteristics were broadly comparable between patients with and without pCR with respect to age, menopausal status, and clinical nodal stage (all P > 0.05). In contrast, patients who achieved pCR had a lower body mass index and higher proliferative activity, as reflected by Ki-67 (both P < 0.001). Tumor burden differed between groups, with a lower proportion of cT3–4 disease and a higher proportion of cT2 tumors observed among patients achieving pCR (P < 0.001). Tumor biological features were also markedly distinct, as ER-negative, PR-negative, and HER2-positive tumors were more frequently observed in the pCR group (all P < 0.001). Total pCR rates differed significantly across the five IHC-defined surrogate subtypes (overall Pearson’s χ² test, P < 0.001). The highest rates were observed in ER-negative/HER2-positive (43.99%) and Luminal B-like/HER2-positive (29.95%) tumors, followed by TNBC (26.47%), whereas Luminal B-like/HER2-negative (10.61%) and Luminal A-like (2.03%) tumors showed markedly lower rates. The three pairwise comparisons displayed in **Figure 1** remained statistically significant after Holm adjustment across all 10 possible pairwise comparisons (all adjusted P < 0.001). Patients achieving pCR were more likely to receive platinum-based chemotherapy and anti-HER2 therapy (both P < 0.001).

**Table 1:** Clinicopathological and neoadjuvant treatment characteristics according to total pathological complete response status.

Variables	Total (n = 3803)	non-pCR (n = 2895)	pCR (n = 908)	P
Age, mean ± SD	49.42 ± 10.33	49.43 ± 10.38	49.42 ± 10.18	0.998
BMI, mean ± SD	24.21 ± 3.34	24.31 ± 3.38	23.90 ± 3.19	<0.001
Ki-67 index (%), mean ± SD	44.01 ± 22.62	41.97 ± 22.50	50.52 ± 21.75	<0.001
Menopause, n (%)				0.263
Premenopausal	2101 (55.25)	1614 (55.75)	487 (53.63)	
Postmenopausal	1702 (44.75)	1281 (44.25)	421 (46.37)	
Clinical T stage, n (%)				<0.001
T1	548 (14.41)	408 (14.09)	140 (15.42)	
T2	2423 (63.71)	1810 (62.52)	613 (67.51)	
T3	587 (15.44)	475 (16.41)	112 (12.33)	
T4	245 (6.44)	202 (6.98)	43 (4.74)	
Clinical N stage, n (%)				0.496
N0	1494 (39.28)	1128 (38.96)	366 (40.31)	
N1	1621 (42.62)	1237 (42.73)	384 (42.29)	
N2	503 (13.23)	394 (13.61)	109 (12.00)	
N3	185 (4.86)	136 (4.70)	49 (5.40)	
ER status, n (%)				<0.001
Negative	1333 (35.05)	854 (29.50)	479 (52.75)	
Positive	2470 (64.95)	2041 (70.50)	429 (47.25)	
PR status, n (%)				<0.001
Negative	1576 (41.44)	1039 (35.89)	537 (59.14)	
Positive	2227 (58.56)	1856 (64.11)	371 (40.86)	
HER2 status, n (%)				<0.001
Negative	2057 (54.09)	1776 (61.35)	281 (30.95)	
Positive	1746 (45.91)	1119 (38.65)	627 (69.05)	
IHC-defined surrogate subtype, n (%)				<0.001
Luminal A-like	246 (6.47)	241 (8.32)	5 (0.55)	
Luminal B-like/HER2−	1282 (33.71)	1146 (39.59)	136 (14.98)	
Luminal B-like/HER2+	1005 (26.43)	704 (24.32)	301 (33.15)	
ER−/HER2+	741 (19.48)	415 (14.34)	326 (35.90)	
TNBC	529 (13.91)	389 (13.44)	140 (15.42)	
Platinum-based chemotherapy, n (%)				<0.001
No	2998 (78.83)	2395 (82.73)	603 (66.41)	
Yes	805 (21.17)	500 (17.27)	305 (33.59)	
Anti-HER2 therapy, n (%)				<0.001
No	2394 (62.95)	2050 (70.81)	344 (37.89)	
Yes	1409 (37.05)	845 (29.19)	564 (62.11)	

BMI, body mass index; ER, estrogen receptor; PR, progesterone receptor; HER2, human epidermal growth factor receptor 2; pCR, pathological complete response. pCR refers to total pathological complete response unless otherwise specified. P values were calculated using appropriate statistical tests. Subtype categories represent IHC-defined clinicopathological surrogate subtypes rather than gene-expression-defined intrinsic subtypes.

**Figure 1:**
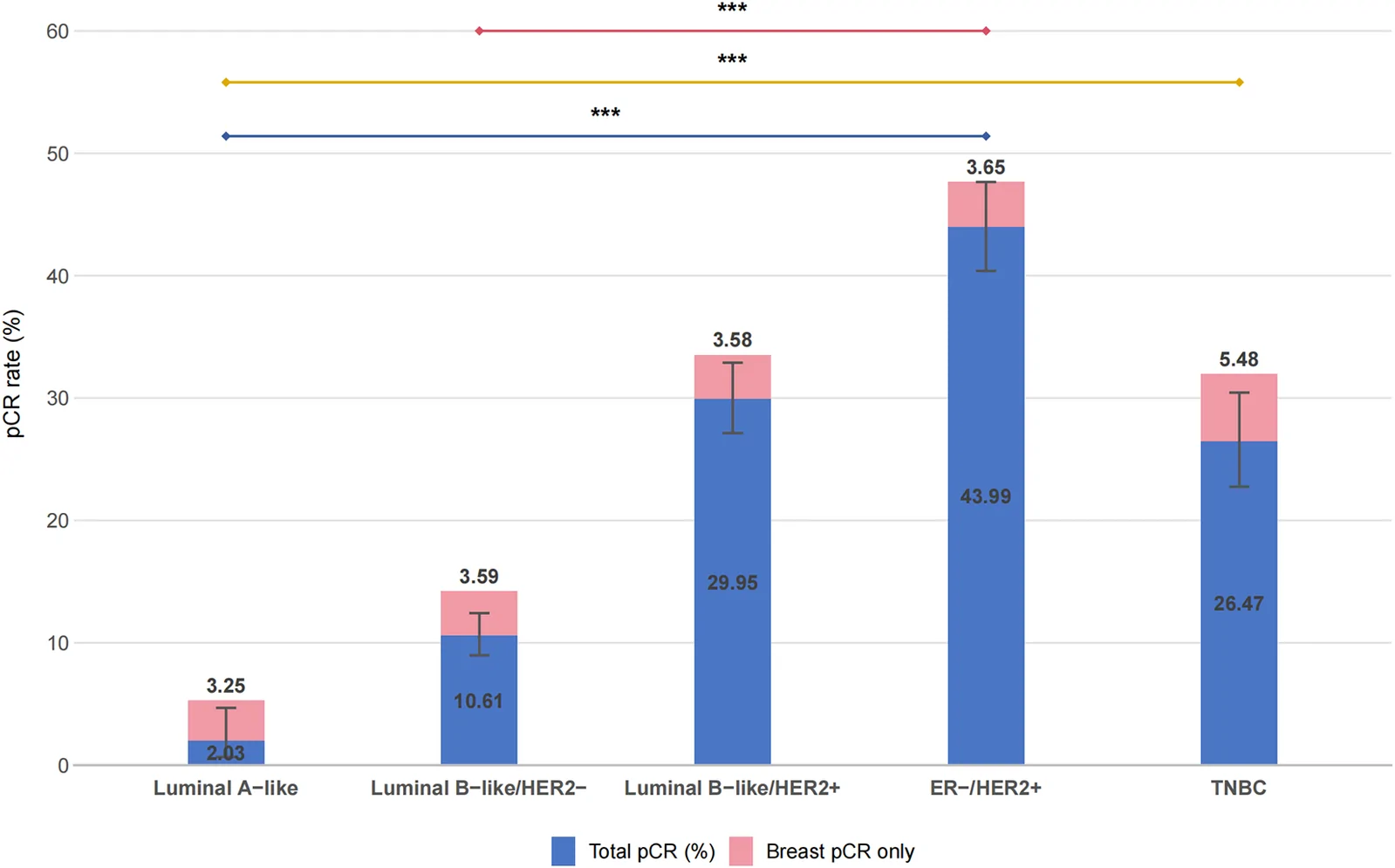
Total and breast pathological complete response rates across IHC-defined surrogate subtypes. Blue segments indicate total pCR (ypT0/is ypN0); pink extensions indicate breast pCR (ypT0/is) with residual nodal disease, so the full bar height represents the breast pCR rate. Error bars show 95% CIs for total pCR. Brackets indicate selected pairwise comparisons; ***P < 0.001 after Holm adjustment over all 10 possible pairwise comparisons. CI, confidence interval; ER, estrogen receptor; HER2, human epidermal growth factor receptor 2; IHC, immunohistochemistry; pCR, pathological complete response; TNBC, triple-negative breast cancer.

### Factors associated with pCR

In adjusted analyses (**Table 2**), IHC-defined surrogate subtype was associated with the likelihood of achieving pCR. Compared with Luminal A-like tumors, ER-negative/HER2-positive tumors (OR 15.82, 95% CI 6.21–40.30) and TNBC (OR 17.07, 95% CI 6.89–42.33) were associated with substantially higher odds of achieving pCR (both P < 0.001). Higher BMI was independently associated with a lower likelihood of pCR (OR 0.97, 95% CI 0.95–1.00, P = 0.021), while increasing tumor burden was inversely related to treatment response, with significantly reduced odds observed in cT3 and cT4 disease. Treatment-related factors also contributed to response, with both platinum-based chemotherapy (OR 1.24, 95% CI 1.03–1.51, P = 0.025) and anti-HER2 therapy (OR 2.71, 95% CI 2.01–3.67, P < 0.001) independently associated with increased pCR rates.

**Table 2:** Factors associated with total pathological complete response: univariable and multivariable logistic regression analyses.

Variables	Univariable analysis		Multivariable analysis	
	OR (95% CI)	P	OR (95% CI)	P
Age	1.00 (0.99–1.01)	0.998	1.00 (0.99–1.00)	0.393
BMI	0.96 (0.94–0.99)	0.001	0.97 (0.95–1.00)	0.021
Menopausal status				
Premenopausal	1.00 (Reference)			
Postmenopausal	1.09 (0.94–1.27)	0.263		
Clinical T stage				
T1	1.00 (Reference)		1.00 (Reference)	
T2	0.99 (0.80–1.22)	0.904	0.93 (0.74–1.17)	0.520
T3	0.69 (0.52–0.91)	0.009	0.61 (0.45–0.82)	0.001
T4	0.62 (0.42–0.91)	0.014	0.58 (0.38–0.87)	0.008
Clinical N stage				
N0	1.00 (Reference)		1.00 (Reference)	
N1	0.96 (0.81–1.13)	0.598	0.93 (0.78–1.11)	0.427
N2	0.85 (0.67–1.09)	0.198	0.83 (0.64–1.07)	0.151
N3	1.11 (0.78–1.57)	0.554	1.04 (0.71–1.51)	0.840
IHC-defined surrogate subtype				
Luminal A-like	1.00 (Reference)		1.00 (Reference)	
Luminal B-like/HER2−	5.72 (2.32–14.11)	<0.001	5.78 (2.34–14.28)	<0.001
Luminal B-like/HER2+	20.61 (8.41–50.47)	<0.001	8.28 (3.25–21.08)	<0.001
ER−/HER2+	37.86 (15.44–92.88)	<0.001	15.82 (6.21–40.30)	<0.001
TNBC	17.35 (7.01–42.94)	<0.001	17.07 (6.89–42.33)	<0.001
Platinum-based chemotherapy				
No	1.00 (Reference)		1.00 (Reference)	
Yes	2.42 (2.05–2.87)	<0.001	1.24 (1.03–1.51)	0.025
Anti-HER2 therapy				
No	1.00 (Reference)		1.00 (Reference)	
Yes	3.98 (3.40–4.65)	<0.001	2.71 (2.01–3.67)	<0.001

OR, odds ratio; CI, confidence interval; BMI, body mass index; HER2, human epidermal growth factor receptor 2; pCR, pathological complete response. pCR refers to total pathological complete response unless otherwise specified. The multivariable model included age, BMI, clinical T stage, clinical N stage, IHC-defined surrogate subtype, platinum-based chemotherapy, and anti-HER2 therapy. ORs and 95% CIs were rounded to two decimal places; two-sided Wald P values were calculated from the unrounded model estimates. Subtype categories represent IHC-defined clinicopathological surrogate subtypes rather than gene-expression-defined intrinsic subtypes. The multivariable logistic regression model included 3,803 patients, of whom 908 achieved total pCR.

In a sensitivity analysis that merged the two HER2-negative luminal categories into a single HR-positive/HER2-negative category, 1,528 patients were classified as HR-positive/HER2-negative, 1,098 as HR-positive/HER2-positive, 648 as HR-negative/HER2-positive, and 529 as TNBC. The corresponding pCR rates were 9.23%, 31.15%, 43.98%, and 26.47%, respectively. Using HR-positive/HER2-negative disease as the reference category, the adjusted odds of achieving pCR were higher for HR-positive/HER2-positive disease (OR 1.74, 95% CI 1.24–2.45; P = 0.001), HR-negative/HER2-positive disease (OR 3.20, 95% CI 2.26–4.53; P < 0.001), and TNBC (OR 3.45, 95% CI 2.66–4.49; P < 0.001) (**Supplementary Table S4**). These findings were directionally consistent with the primary five-category analysis under a broader receptor-based classification that did not retain the Ki-67- and PR-based distinction between the two HER2-negative luminal categories.

### Treatment patterns over the study period

Treatment patterns varied over the 2012–2021 study period (**Supplementary Table S3**). The most frequently recorded structured neoadjuvant chemotherapy labels were EC-T (778/3,803, 20.5%), TEC (336/3,803, 8.8%), TCbH (236/3,803, 6.2%), and EC-TH (187/3,803, 4.9%); less frequent structured labels included EC, TCb, and PCb (**Supplementary Table S3**). However, 1,809 patients (47.6%) were recorded under heterogeneous non-standardized free-text regimens, and the structured regimen label was unavailable for 25 patients (0.7%). Accordingly, regimen-level chemotherapy backbone and sequencing were summarized descriptively rather than analyzed as standardized covariates. Among the 1,409 patients who received anti-HER2 therapy, 663 (47.1%) received trastuzumab alone, 646 (45.8%) received trastuzumab plus pertuzumab, 98 (7.0%) received trastuzumab plus pyrotinib, and 2 (0.1%) received pyrotinib alone. Broad postoperative treatment indicators were complete for the analytic cohort. Overall, 969 patients (25.5%) were recorded as receiving adjuvant chemotherapy, 2,823 (74.2%) as receiving radiotherapy, and 2,106 (55.4%) as receiving endocrine therapy. Among patients without pCR and those with pCR, respectively, the corresponding frequencies were 28.7% and 15.1% for adjuvant chemotherapy, 74.9% and 72.0% for radiotherapy, and 60.6% and 38.9% for endocrine therapy (**Supplementary Table S3**). Within the ER-negative/HER2-positive subgroup (n = 741), 607 patients (81.9%) received anti-HER2 therapy; among these, 280 (46.1%) received trastuzumab alone, 290 (47.8%) received trastuzumab plus pertuzumab, 36 (5.9%) received trastuzumab plus pyrotinib, and 1 (0.2%) received pyrotinib alone. Among the 415 ER-negative/HER2-positive patients who did not achieve pCR, 106 (25.5%) were recorded as receiving adjuvant chemotherapy. However, regimen-level post-neoadjuvant HER2-directed treatment was not available in the analytic dataset; therefore, whether residual disease led to specific HER2-directed treatment escalation could not be reliably determined. Among the 389 patients with TNBC who did not achieve pCR, 215 (55.3%) were recorded as receiving adjuvant chemotherapy. However, regimen-level postoperative chemotherapy information was not available in the analytic dataset; therefore, the use of specific agents such as capecitabine could not be reliably determined. Among 2,626 patients with ER and/or PR-positive disease, 2,024 (77.1%) were recorded as receiving endocrine therapy. However, specific endocrine agents, treatment duration, treatment modifications, and adherence were not available in the analytic dataset. The use of dual anti-HER2 blockade increased markedly during the later years of the study period. Because chemotherapy regimen coding was heterogeneous in the analytic dataset and treatment-cycle information was not consistently available, individual chemotherapy regimens and cycle numbers were not incorporated as standardized covariates in the regression models.

In sensitivity analyses additionally adjusting for calendar year of surgery as a categorical covariate, the associations between IHC-defined surrogate subtype and the likelihood of achieving pCR remained materially unchanged. Anti-HER2 therapy remained independently associated with a higher likelihood of pCR (OR 2.38, 95% CI 1.76–3.26; P < 0.001), whereas the association between platinum-based chemotherapy and pCR was attenuated and was no longer statistically significant (OR 1.10, 95% CI 0.90–1.33; P = 0.361). The associations of pCR with RFS and BCSS remained materially similar after adjustment for calendar year of surgery (RFS: HR 0.38, 95% CI 0.24–0.59; P < 0.001; BCSS: HR 0.32, 95% CI 0.19–0.53; P < 0.001).

### Association between pCR and survival

During a median follow-up of 46 months (interquartile range, 35–60 months), 274 RFS events and 239 breast cancer-specific deaths were observed in the overall cohort. Among the 2,895 patients without pCR, 251 experienced an RFS event and 222 experienced a breast cancer-specific death, whereas among the 908 patients who achieved pCR, the corresponding numbers were 23 and 17, respectively. Patients who achieved pCR therefore experienced a markedly reduced risk of recurrence or death and breast cancer-specific mortality compared with those without pCR, and this association was consistently observed across crude and adjusted analyses (**Figure 2**). In the exploratory propensity-score-matched analysis, 907 of 908 patients who achieved pCR were matched 1:1 to 907 of 2,895 patients who did not achieve pCR, yielding 907 matched pairs (n = 1,814); one patient with pCR and 1,988 patients without pCR were not included in the matched cohort. Covariate balance improved substantially after matching, with all prespecified covariates achieving absolute standardized mean differences <0.10 (**Supplementary Table S1**; **Supplementary Figure S1**). The matched cohort included 77 RFS events and 63 breast cancer-specific deaths. In matched-pair robust Cox analyses, pCR remained associated with improved RFS (HR 0.43, 95% CI 0.27–0.69; P < 0.001) and BCSS (HR 0.38, 95% CI 0.22–0.64; P < 0.001) (**Figure 3**). Results were also consistent in the overlap-weighted sensitivity analysis. The analysis retained all 3,803 observations and yielded an overall effective sample size of 2,327.2. After weighting, all modeled covariates had absolute weighted SMDs <0.001, showing numerical balance for those recorded variables (**Supplementary Table S1**). pCR remained associated with improved RFS (HR 0.38, 95% CI 0.24–0.60; P < 0.001) and BCSS (HR 0.32, 95% CI 0.19–0.54; P < 0.001). In multivariable analyses adjusting for clinicopathological factors (**Tables 3** and **4**), pCR remained associated with a lower hazard of recurrence or death from any cause (HR 0.37, 95% CI 0.24–0.57; P < 0.001) and breast cancer-specific death (HR 0.30, 95% CI 0.18–0.50; P < 0.001). Increasing tumor burden and nodal involvement were associated with worse outcomes, whereas anti-HER2 therapy was consistently associated with improved survival. Platinum-based chemotherapy did not retain statistical significance after adjustment. IHC-defined surrogate subtype remained an independent prognostic factor, with non-Luminal A-like surrogate subtypes generally associated with higher risks. These subtype main-effect estimates describe differences in overall prognosis relative to the Luminal A-like reference category and do not test whether the association between pCR and survival differs across subtypes. In the primary Cox models, Schoenfeld residual tests provided no evidence of non-proportional hazards for pCR (RFS, P = 0.105; BCSS, P = 0.219), and the global tests were not statistically significant for either RFS (P = 0.095) or BCSS (P = 0.112). However, the IHC-defined surrogate subtype term showed evidence of non-proportional hazards (RFS, P = 0.020; BCSS, P = 0.015). In sensitivity analyses using Cox models stratified by IHC-defined surrogate subtype, the associations between pCR and survival remained essentially unchanged for RFS (HR 0.37, 95% CI 0.24–0.57; P < 0.001) and BCSS (HR 0.30, 95% CI 0.18–0.50; P < 0.001). Global Schoenfeld residual tests provided no evidence of non-proportional hazards in the subtype-stratified models (RFS, P = 0.462; BCSS, P = 0.596). The survival sensitivity analyses are summarized in **Supplementary Table S2**.

**Figure 2:**
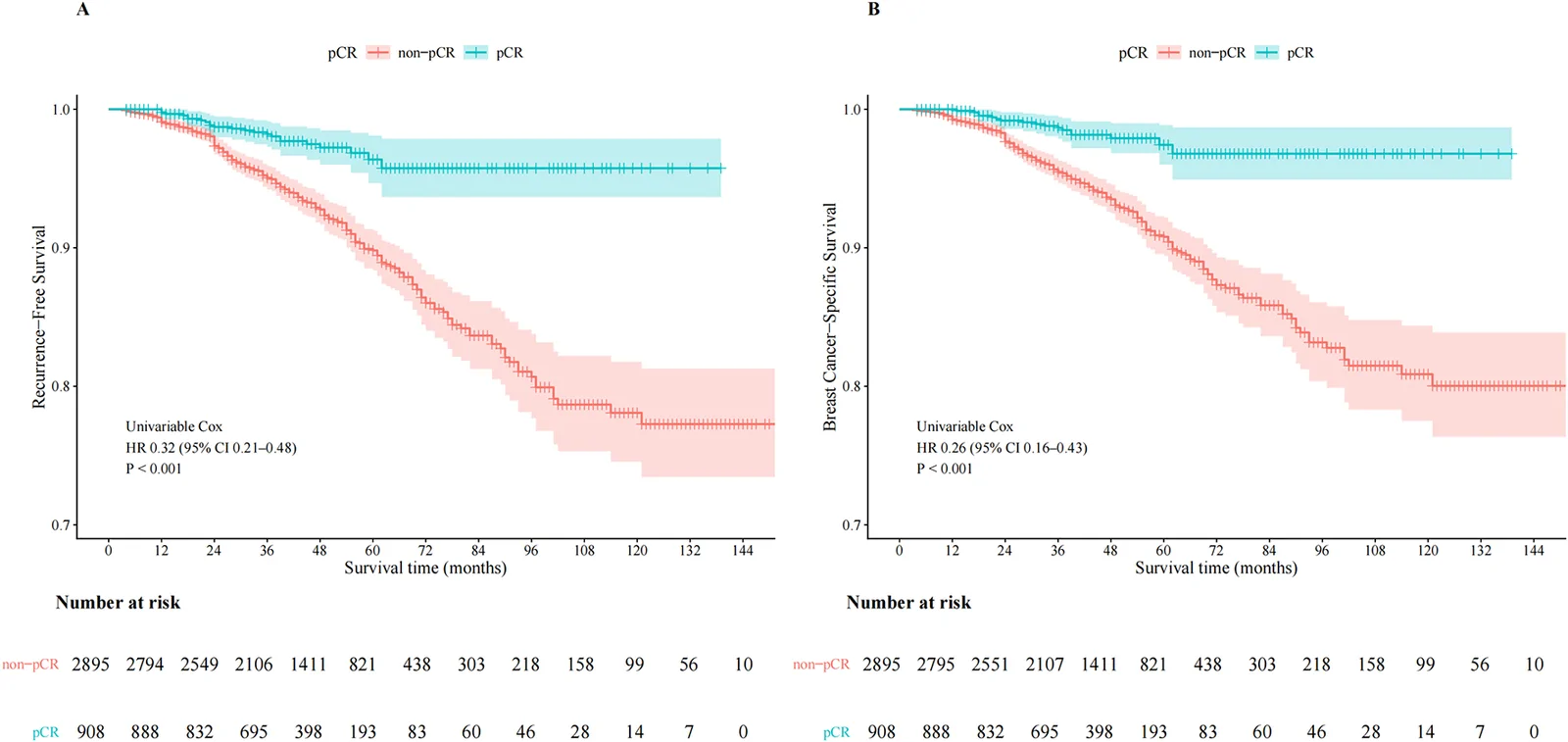
Recurrence-free and breast cancer-specific survival by total pathological complete response status in the overall cohort. Kaplan–Meier curves show **(A)** RFS and **(B)** BCSS; shaded bands indicate 95% CIs and tick marks indicate censoring. Displayed HRs, 95% CIs, and two-sided Wald P values are from univariable Cox models comparing pCR with non-pCR (reference). Total pCR was defined as ypT0/is ypN0. BCSS, breast cancer-specific survival; CI, confidence interval; HR, hazard ratio; pCR, pathological complete response; RFS, recurrence-free survival.

**Figure 3:**
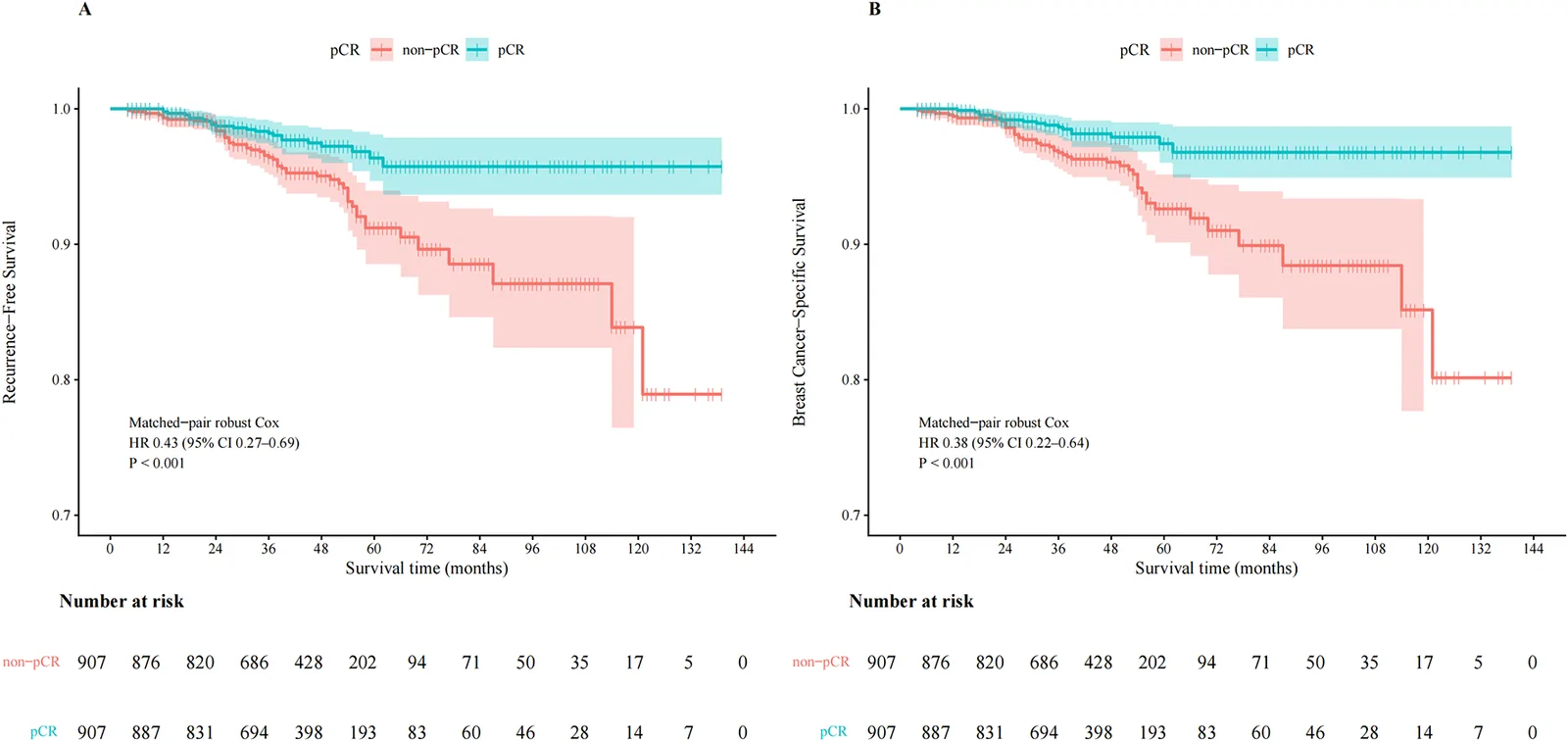
Recurrence-free and breast cancer-specific survival by total pathological complete response status in the propensity score-matched cohort. After 1:1 matching (907 pairs), Kaplan–Meier curves show **(A)** RFS and **(B)** BCSS; shaded bands indicate 95% CIs and tick marks indicate censoring. Displayed HRs, 95% CIs, and two-sided Wald P values are from Cox models with robust standard errors clustered by matched pair, comparing pCR with non-pCR (reference). Total pCR was defined as ypT0/is ypN0. BCSS, breast cancer-specific survival; CI, confidence interval; HR, hazard ratio; pCR, pathological complete response; RFS, recurrence-free survival.

**Table 3:** Factors associated with recurrence-free survival: univariable and multivariable Cox regression analyses.

Variables	Univariable analysis		Multivariable analysis	
	HR (95% CI)	P	HR (95% CI)	P
pCR				
No	1.00 (Reference)		1.00 (Reference)	
Yes	0.32 (0.21–0.48)	<0.001	0.37 (0.24–0.57)	<0.001
Age	1.00 (0.99–1.01)	0.794	0.99 (0.98–1.01)	0.368
BMI	1.01 (0.97–1.04)	0.662	1.00 (0.97–1.04)	0.788
Menopausal status				
Premenopausal	1.00 (Reference)			
Postmenopausal	1.09 (0.86–1.38)	0.474		
Clinical T stage				
T1	1.00 (Reference)		1.00 (Reference)	
T2	1.21 (0.81–1.81)	0.356	1.27 (0.85–1.91)	0.249
T3	2.23 (1.43–3.47)	<0.001	2.09 (1.34–3.26)	0.001
T4	3.12 (1.89–5.15)	<0.001	2.81 (1.69–4.67)	<0.001
Clinical N stage				
N0	1.00 (Reference)		1.00 (Reference)	
N1	1.54 (1.15–2.06)	0.003	1.44 (1.08–1.93)	0.013
N2	1.88 (1.31–2.68)	<0.001	1.65 (1.15–2.37)	0.007
N3	3.24 (2.08–5.03)	<0.001	2.83 (1.81–4.43)	<0.001
IHC-defined surrogate subtype				
Luminal A-like	1.00 (Reference)		1.00 (Reference)	
Luminal B-like/HER2−	3.67 (1.71–7.88)	<0.001	3.91 (1.82–8.42)	<0.001
Luminal B-like/HER2+	1.70 (0.76–3.81)	0.195	4.09 (1.77–9.46)	<0.001
ER−/HER2+	2.51 (1.13–5.56)	0.023	6.61 (2.87–15.19)	<0.001
TNBC	5.05 (2.32–11.02)	<0.001	5.97 (2.72–13.09)	<0.001
Platinum-based chemotherapy				
No	1.00 (Reference)		1.00 (Reference)	
Yes	0.70 (0.50–0.99)	0.046	0.97 (0.67–1.40)	0.864
Anti-HER2 therapy				
No	1.00 (Reference)		1.00 (Reference)	
Yes	0.37 (0.27–0.51)	<0.001	0.36 (0.23–0.56)	<0.001

HR, hazard ratio; CI, confidence interval; BMI, body mass index; HER2, human epidermal growth factor receptor 2; pCR, pathological complete response; RFS, recurrence-free survival. pCR refers to total pathological complete response unless otherwise specified. The multivariable Cox model was adjusted for age, BMI, clinical T stage, clinical N stage, IHC-defined surrogate subtype, platinum-based chemotherapy, and anti-HER2 therapy. Tied event times were handled using the Efron approximation; 95% confidence intervals and two-sided P values were Wald based. Displayed HRs and confidence intervals were rounded to two decimal places; P values were calculated from the unrounded model estimates. Subtype categories represent IHC-defined clinicopathological surrogate subtypes rather than gene-expression-defined intrinsic subtypes. The multivariable Cox model included 3,803 patients and 274 RFS events.

**Table 4:** Factors associated with breast cancer-specific survival: univariable and multivariable Cox regression analyses.

Variables	Univariable analysis		Multivariable analysis	
	HR (95% CI)	P	HR (95% CI)	P
pCR				
No	1.00 (Reference)		1.00 (Reference)	
Yes	0.26 (0.16–0.43)	<0.001	0.30 (0.18–0.50)	<0.001
Age	0.99 (0.98–1.01)	0.310	0.99 (0.98–1.00)	0.083
BMI	1.01 (0.98–1.05)	0.442	1.01 (0.98–1.05)	0.497
Menopausal status				
Premenopausal	1.00 (Reference)			
Postmenopausal	0.99 (0.77–1.28)	0.944		
Clinical T stage				
T1	1.00 (Reference)		1.00 (Reference)	
T2	1.02 (0.68–1.54)	0.922	1.09 (0.72–1.64)	0.691
T3	1.93 (1.23–3.03)	0.004	1.79 (1.14–2.82)	0.012
T4	2.86 (1.72–4.77)	<0.001	2.57 (1.53–4.32)	<0.001
Clinical N stage				
N0	1.00 (Reference)		1.00 (Reference)	
N1	1.45 (1.06–1.98)	0.019	1.37 (1.00–1.88)	0.047
N2	2.07 (1.43–3.00)	<0.001	1.81 (1.24–2.63)	0.002
N3	3.29 (2.06–5.25)	<0.001	2.90 (1.80–4.67)	<0.001
IHC-defined surrogate subtype				
Luminal A-like	1.00 (Reference)		1.00 (Reference)	
Luminal B-like/HER2−	3.10 (1.44–6.68)	0.004	3.36 (1.55–7.26)	0.002
Luminal B-like/HER2+	1.43 (0.63–3.24)	0.387	3.59 (1.53–8.40)	0.003
ER−/HER2+	2.29 (1.03–5.09)	0.042	6.43 (2.77–14.91)	<0.001
TNBC	4.44 (2.02–9.72)	<0.001	5.40 (2.45–11.91)	<0.001
Platinum-based chemotherapy				
No	1.00 (Reference)		1.00 (Reference)	
Yes	0.70 (0.48–1.01)	0.055	0.97 (0.65–1.44)	0.867
Anti-HER2 therapy				
No	1.00 (Reference)		1.00 (Reference)	
Yes	0.37 (0.27–0.52)	<0.001	0.35 (0.22–0.57)	<0.001

HR, hazard ratio; CI, confidence interval; BMI, body mass index; HER2, human epidermal growth factor receptor 2; pCR, pathological complete response; BCSS, breast cancer-specific survival. pCR refers to total pathological complete response unless otherwise specified. The multivariable Cox model was adjusted for age, BMI, clinical T stage, clinical N stage, IHC-defined surrogate subtype, platinum-based chemotherapy, and anti-HER2 therapy. Tied event times were handled using the Efron approximation; 95% confidence intervals and two-sided P values were Wald based. Displayed HRs and confidence intervals were rounded to two decimal places; P values were calculated from the unrounded model estimates. Subtype categories represent IHC-defined clinicopathological surrogate subtypes rather than gene-expression-defined intrinsic subtypes. The multivariable Cox model included 3,803 patients and 239 breast cancer-specific deaths.

### Exploratory subgroup analyses by IHC-defined surrogate subtype

Subgroup-specific estimates of the association between pCR and survival outcomes varied numerically across the subtypes with estimable within-subtype contrasts (**Figure 4**). The Luminal A-like subgroup comprised 246 patients, of whom only five achieved pCR. None of these five patients experienced an RFS event or breast cancer-specific death, whereas seven RFS events and seven breast cancer-specific deaths occurred among the 241 patients without pCR. Consequently, the subgroup-specific hazard ratios for Luminal A-like disease were not estimable, and the conventional five-level pCR-by-subtype interaction models exhibited monotone likelihood and did not permit reliable standard inference. Exploratory covariate-adjusted global likelihood-ratio tests were therefore restricted to the remaining four subtypes with estimable within-subtype pCR contrasts. These tests showed no statistical evidence of heterogeneity for either RFS (likelihood-ratio χ² = 1.576, df = 3, P for interaction = 0.665) or BCSS (likelihood-ratio χ² = 1.349, df = 3, P for interaction = 0.718).

**Figure 4:**
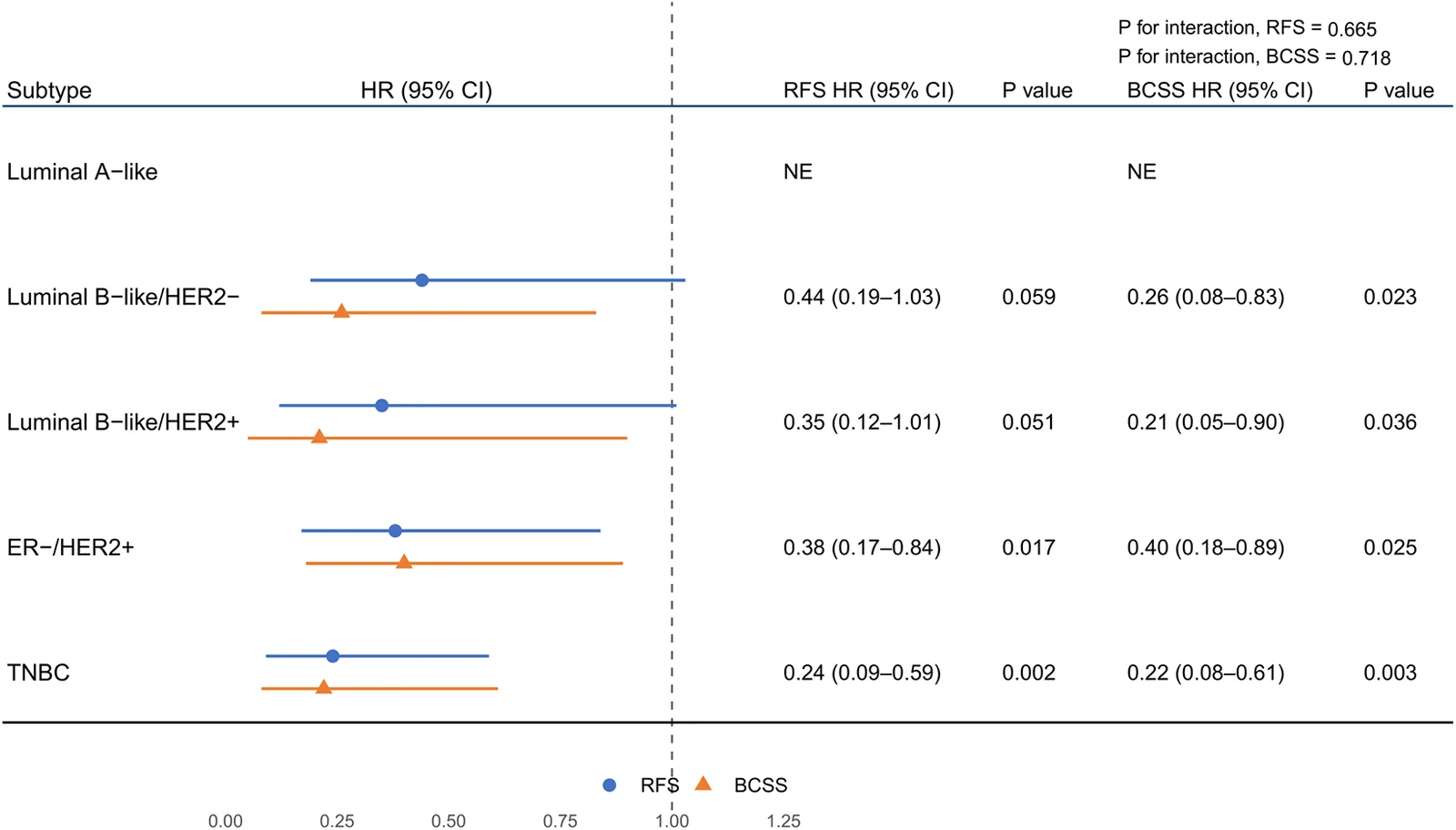
Exploratory associations of total pathological complete response with survival across IHC-defined surrogate subtypes. Analyses used the original unmatched and unweighted cohort. Within-subtype HRs, 95% CIs, and two-sided Wald P values compare pCR with non-pCR (reference) in Cox models adjusted for age, body mass index, clinical T and N stage, platinum-based chemotherapy, and anti-HER2 therapy; ties were handled using the Efron method. Global pCR-by-subtype interaction was evaluated among the four estimable subtypes (all except Luminal A-like) using 3-df likelihood-ratio tests with the same adjustment and tie handling; there was no statistical evidence of heterogeneity for RFS (χ² = 1.576, P for interaction = 0.665) or BCSS (χ² = 1.349, P for interaction = 0.718). Luminal A-like was excluded because none of its five patients with pCR experienced an RFS or BCSS event; its HR is shown as NE, indicating non-estimability rather than absence of effect. Total pCR was defined as ypT0/is ypN0. BCSS, breast cancer-specific survival; CI, confidence interval; df, degrees of freedom; ER, estrogen receptor; HER2, human epidermal growth factor receptor 2; HR, hazard ratio; IHC, immunohistochemistry; NE, not estimable; pCR, pathological complete response; RFS, recurrence-free survival; TNBC, triple-negative breast cancer.

The conventional four-subtype interaction models included 3,557 patients, with 267 RFS events and 232 breast cancer-specific deaths, and converged without warnings. Schoenfeld residual tests provided no evidence of non-proportional hazards for subtype (RFS, P = 0.315; BCSS, P = 0.280), the pCR-by-subtype interaction (RFS, P = 0.447; BCSS, P = 0.620), or globally (RFS, P = 0.547; BCSS, P = 0.695). In sensitivity analyses allowing subtype-specific baseline hazards, the interaction findings remained nonsignificant for RFS (likelihood-ratio χ² = 1.831, df = 3, P = 0.608) and BCSS (likelihood-ratio χ² = 1.720, df = 3, P = 0.633) (**Supplementary Table S7**).

These interaction tests do not apply to Luminal A-like disease. Among the four included subtypes, the nonsignificant interaction results do not prove that the pCR–survival associations are identical. Conversely, neither numerical differences in subtype-specific HRs nor a statistically significant association in one subgroup but not another should be interpreted as establishing effect modification. Accordingly, the subtype-specific estimates should be regarded as descriptive, exploratory, and hypothesis-generating.

Complete event counts by subtype and pCR status and model diagnostics are provided in **Supplementary Table S6**. Across the eight endpoint-specific models for the four subtypes with estimable pCR contrasts, each model included 10 or 11 fitted regression parameters; no convergence warnings occurred, and all fitted coefficients and standard errors were finite. However, only 4–8 RFS events and 2–8 breast cancer-specific deaths occurred among patients with pCR within these subtypes, limiting the precision and stability of the adjusted estimates. In Luminal A-like disease, the absence of events among the five patients with pCR produced a monotone-likelihood warning and precluded estimation of the pCR HR.

## Discussion

In this large real-world cohort derived from a multicenter database, achievement of pCR after neoadjuvant therapy was associated with favorable RFS and BCSS. The principal contribution of the present study is not the observation that pCR is prognostic per se, which has been established in previous pooled and meta-analytic studies (3, 4), but the evaluation of this association within a large multicenter database using complementary survival endpoints and multiple analytical approaches. Specifically, we simultaneously examined determinants of pCR across IHC-defined surrogate subtypes, performed exploratory global interaction testing among the four subtypes with estimable within-subtype pCR contrasts, and assessed the consistency of the overall prognostic association using exploratory covariate-balancing analyses based on propensity score matching and overlap weighting, treatment-era adjustment, and subtype-stratified Cox models. Across these complementary analyses, the overall association between pCR and favorable long-term outcomes remained consistent.

Beyond the overall prognostic association of pCR, we observed marked differences in the frequency of pCR across IHC-defined surrogate subtypes, consistent with established differences in tumor biology and treatment sensitivity (13, 14). A recent Brazilian real-world cohort also evaluated pCR and survival outcomes across breast cancer subtypes after neoadjuvant chemotherapy (15). Subgroup-specific hazard ratios for RFS and BCSS also varied numerically. However, because only five patients with Luminal A-like disease achieved pCR and none experienced an RFS or BCSS event, the conventional five-level pCR-by-subtype interaction models exhibited monotone likelihood and did not permit reliable standard inference. Exploratory global likelihood-ratio tests restricted to the remaining four subtypes with estimable within-subtype pCR contrasts showed no statistical evidence of heterogeneity for RFS (P for interaction = 0.665) or BCSS (P for interaction = 0.718). The exclusion of Luminal A-like disease was based solely on estimability rather than on the magnitude or statistical significance of a subgroup result. Nevertheless, these tests do not address Luminal A-like disease. Among the four included subtypes, the absence of a statistically significant interaction does not prove identical pCR–survival associations. Conversely, neither numerical differences in subtype-specific point estimates nor a statistically significant association in one subgroup but not another should be interpreted as establishing effect modification, particularly given the limited number of events in some subgroups (16). The subtype-specific findings should therefore be interpreted as exploratory and hypothesis-generating rather than confirmatory, and adequately powered studies are required to determine whether the prognostic association of pCR truly differs across IHC-defined surrogate subtypes.

An additional consideration is the emerging clinical relevance of HER2-low disease, conventionally defined as HER2 IHC 1+ or IHC 2+ with negative *in situ* hybridization. The 2023 ASCO–College of American Pathologists guideline update affirmed the existing HER2 testing framework and did not establish a new formal HER2-low interpretive category, while acknowledging the clinical relevance of distinguishing IHC 0 from IHC 1+ in the context of eligibility for trastuzumab deruxtecan (17). HER2-low should therefore be viewed here as an operational, treatment-relevant HER2-expression category rather than an established gene-expression-defined intrinsic subtype. In the present study, HER2 was analyzed using the conventional HER2-positive versus HER2-negative framework, and HER2-low and HER2-zero tumors were not evaluated as separate prespecified categories. This distinction has become increasingly relevant with the development of antibody–drug conjugates, particularly trastuzumab deruxtecan, which has demonstrated substantial clinical benefit in HER2-low advanced breast cancer (18). In the neoadjuvant setting, a pooled individual-patient analysis of 2,310 participants from four prospective trials found a lower pCR rate for HER2-low than HER2-zero tumors overall (29.2% vs 39.0%), with this difference observed in the hormone receptor-positive subgroup but not in the hormone receptor-negative subgroup (19). Subsequent meta-analytic and real-world studies have likewise described differences in pCR and survival outcomes between HER2-low and HER2-zero disease, although their magnitude and prognostic implications remain incompletely defined (20, 21). Accordingly, the HER2-negative groups in our cohort may contain biologically and clinically heterogeneous HER2-low and HER2-zero tumors, and the present analysis cannot determine whether low-level HER2 expression modifies the probability or prognostic significance of pCR. Future real-world studies incorporating granular HER2 IHC and ISH results should evaluate HER2-low and emerging HER2-ultralow categories more specifically.

Clinically, the interpretation of pCR and residual disease must be considered in the context of subsequent systemic therapy. In HER2-positive disease, the post-neoadjuvant treatment landscape has evolved substantially. The KATHERINE trial established T-DM1 as an effective post-neoadjuvant treatment for patients with residual invasive disease after trastuzumab-based neoadjuvant therapy, with durable invasive disease-free and overall survival benefits demonstrated on long-term follow-up (22, 23). More recently, DESTINY-Breast05 directly compared trastuzumab deruxtecan (T-DXd) with T-DM1 in patients with high-risk residual HER2-positive early breast cancer. T-DXd significantly improved invasive disease-free survival, with a hazard ratio of 0.47 (95% CI 0.34–0.66) and 3-year invasive disease-free survival rates of 92.4% versus 83.7% with T-DM1 (24). These findings further redefine the clinical significance of residual HER2-positive disease and demonstrate that the prognostic implications of non-pCR are increasingly influenced by highly effective response-adapted post-neoadjuvant therapy. In DESTINY-Breast11, neoadjuvant T-DXd followed by paclitaxel, trastuzumab, and pertuzumab achieved a higher pCR rate than dose-dense doxorubicin and cyclophosphamide followed by paclitaxel, trastuzumab, and pertuzumab in high-risk HER2-positive early breast cancer (25). Contemporary real-world evidence has likewise shown that neoadjuvant trastuzumab plus pertuzumab with chemotherapy can achieve substantial pCR rates outside randomized trials, with the NEOPERSUR study reporting an overall pCR rate of 62.2% and higher pCR rates among patients with hormone receptor-negative tumors and tumors with Ki-67 >20% (26). Because our cohort was treated between 2012 and 2021 and regimen-level post-neoadjuvant HER2-directed therapy was not captured, our HER2-positive survival estimates largely reflect an earlier treatment era and should not be interpreted as directly representing outcomes under contemporary T-DXd-based treatment strategies.

In TNBC, adjuvant capecitabine is an evidence-based post-neoadjuvant option for patients with residual invasive disease after anthracycline- and/or taxane-containing neoadjuvant chemotherapy, as supported by findings in the TNBC subgroup of the CREATE-X trial (27). More recently, the treatment paradigm for high-risk early TNBC has evolved from chemotherapy alone toward perioperative chemoimmunotherapy. In KEYNOTE-522, the addition of pembrolizumab to neoadjuvant platinum-containing chemotherapy, followed by continuation of pembrolizumab after surgery, increased pCR rates and improved event-free survival; with longer follow-up, this strategy also resulted in a significant overall survival benefit (28, 29). A preplanned exploratory analysis further showed that pembrolizumab shifted patients toward lower residual cancer burden categories and was associated with fewer event-free survival events in the RCB-0, RCB-I, and RCB-II groups, with the greatest relative benefit in RCB-II (30). A contemporary real-world cohort likewise reported a higher pCR rate with the KEYNOTE-522 regimen than in the comparison cohort treated with neoadjuvant therapy without pembrolizumab (59.3% vs 33.1%). Grade 2 or higher immune-related adverse events occurred in 34.9% of patients receiving the KEYNOTE-522 regimen; at a median follow-up of 2.4 years, no statistically significant difference in survival outcomes was detected (31). These findings place pathological response within a substantially different contemporary treatment context from that represented by much of our cohort. Because patients in the present study were treated between 2012 and 2021, and regimen-level immunotherapy information was not available in the analytic dataset, our TNBC cohort largely reflects an era before perioperative pembrolizumab became routinely incorporated into the treatment of high-risk early TNBC. The prognostic estimates for pCR in TNBC should therefore be interpreted in the context of this treatment-era difference.

For patients with germline BRCA1/2 pathogenic or likely pathogenic variants and high-risk HER2-negative early breast cancer, the OlympiA trial demonstrated improved invasive disease-free and distant disease-free survival with 1 year of adjuvant olaparib after completion of local treatment and neoadjuvant or adjuvant chemotherapy (32). For patients treated with neoadjuvant chemotherapy, eligibility required residual invasive disease; those with hormone receptor-positive disease additionally required a CPS+EG score of at least 3. Because standardized germline BRCA1/2 testing results and olaparib-use information were not available in the analytic dataset, their influence on outcomes could not be evaluated.

In hormone receptor-positive disease, long-term outcomes are additionally influenced by postoperative endocrine therapy. Information on endocrine therapy was limited to broad treatment indicators, without sufficiently standardized details on specific agents, duration, treatment modification, or adherence. These limitations are important because post-neoadjuvant treatment decisions may influence subsequent survival and may differ according to pathological response and tumor subtype.

Together, these studies show that effective post-neoadjuvant therapy can improve outcomes in patients with residual disease without changing their already established pathological response status (22–24, 27, 32). The pCR–survival association observed in this cohort should therefore be interpreted within the treatment context represented by the 2012–2021 study period. Its magnitude cannot be assumed to remain unchanged under contemporary response-guided treatment. Our data cannot determine whether such treatment narrows or otherwise changes the outcome difference between patients with and without pCR, and the observed HRs do not provide evidence for selecting or omitting a specific postoperative therapy.

From a methodological perspective, the prognostic value of pCR at the individual-patient level should be distinguished from its validity as a trial-level surrogate endpoint. At the individual-patient level, achievement of pCR is consistently associated with a lower subsequent risk of recurrence and death, and the present study provides additional real-world evidence supporting this prognostic association. Trial-level surrogacy addresses a different question: whether the magnitude of treatment-induced improvement in pCR rates reliably predicts the magnitude of improvement in long-term outcomes such as disease-free survival, event-free survival, or overall survival across randomized treatment comparisons. Meta-analytic evidence indicates that these trial-level associations are weak despite the strong patient-level prognostic association of pCR (4, 33). Accordingly, regulatory use of pCR as a potential endpoint to support accelerated approval should not be interpreted as evidence that an increase in pCR rate necessarily translates into a proportional improvement in long-term survival (5, 33). Our observational analysis evaluates pCR as an individual-patient prognostic marker and does not assess or validate pCR as a trial-level surrogate endpoint.

Beyond the binary distinction between pCR and non-pCR, residual cancer burden (RCB) provides a more granular assessment of pathological response by integrating the extent and cellularity of residual disease in the breast with residual nodal involvement. RCB can be expressed as a continuous score and categorized into RCB-0 (pCR), RCB-I, RCB-II, and RCB-III, thereby capturing substantial prognostic heterogeneity among patients who do not achieve pCR (34, 35). In a multicenter pooled analysis of 5,161 patients treated with neoadjuvant chemotherapy, Yau et al. demonstrated that increasing RCB was associated with progressively worse event-free survival and that the prognostic relevance of RCB was evident across breast cancer phenotypic subtypes (35). These findings illustrate an important limitation of a binary pCR/non-pCR endpoint: patients with residual disease constitute a heterogeneous population with markedly different levels of residual risk. RCB may therefore complement pCR by providing more refined post-neoadjuvant prognostic stratification and may be particularly informative when identifying patients with residual disease who remain at high risk of recurrence. RCB was not available in a sufficiently standardized form in our analytic dataset and could not be retrospectively reconstructed; consequently, the present study was restricted to the predefined binary pCR endpoint. Exploratory analyses of serial circulating tumor DNA during neoadjuvant therapy have linked molecular response with pathological response and subsequent outcomes (36). Future real-world studies integrating pCR, RCB, circulating tumor DNA, genomic risk, and other dynamic biomarkers may provide a more comprehensive framework for post-neoadjuvant risk assessment.

Several limitations should be acknowledged. This retrospective cohort included only patients who reached surgery and may be affected by source-level missing-data selection and residual treatment, center, and access-related confounding (37, 38). Unavailable center identifiers precluded accounting for center effects; positive within-center correlation could have narrowed confidence intervals. Because pCR is a response state, propensity-score analyses describe associations rather than causal effects, and response-guided postoperative therapy may influence these associations. IHC-defined surrogate classification and incompletely documented Ki-67 assessment may introduce misclassification (10, 11), while the median follow-up of 46 months may underrepresent late hormone receptor-positive recurrence (39). For BCSS, non-breast cancer death indicators and times were unavailable and their censoring rule was unverifiable, precluding competing-risk analysis; a separate loss-to-follow-up rate could not be quantified. Sparse events precluded reliable estimation of the pCR association and interaction testing in Luminal A-like disease and limited the remaining subtype-specific estimates, which remain exploratory.

Taken together, our findings show substantial differences in pCR frequency across IHC-defined surrogate subtypes and support an overall association between pCR and favorable RFS and BCSS in this cohort. Among the four subtypes with estimable within-subtype pCR contrasts, exploratory interaction tests did not provide statistical evidence of heterogeneity; Luminal A-like disease could not be assessed because of sparse events. These findings should not be interpreted as proving a uniform prognostic association across all subtypes, and the subtype-specific estimates remain exploratory and hypothesis-generating. Future adequately powered studies with longer follow-up and granular information on residual disease burden, genomic risk, response-adapted therapy, and dynamic biomarkers are needed to determine whether IHC-defined surrogate subtype modifies the prognostic association of pCR and to refine post-neoadjuvant risk stratification.

## Data Availability

The raw data supporting the conclusions of this article will be made available by the authors, without undue reservation.
