# Supplementary material for: Pathological complete response and long-term outcomes after neoadjuvant therapy for breast cancer: a multicenter retrospective cohort study with exploratory subtype analyses
Source: Front Oncol. 2026 Oct 23;16:1994688. doi: 10.3389/fonc.2026.1994688 (PMC13739544; doi:10.3389/fonc.2026.1994688)
Supplement: Supplementary file 1 [file DataSheet1.docx]

**Supplementary Table S1. Covariate balance before and after propensity score matching and overlap weighting**

| **Variable** | **Before PSM** | | | **After PSM** | | | **After overlap weighting** |
| --- | --- | --- | --- | --- | --- | --- | --- |
|  | **non-pCR (n = 2895)** | **pCR (n = 908)** | **Absolute SMD** | **non-pCR (n = 907)** | **pCR (n = 907)** | **Absolute SMD** | **Absolute weighted SMD** |
| Age, years | 49.43 ± 10.38 | 49.42 ± 10.18 | 0.000 | 49.38 ± 10.50 | 49.44 ± 10.17 | 0.006 | <0.001 |
| BMI, kg/m² | 24.31 ± 3.38 | 23.90 ± 3.19 | 0.128 | 23.93 ± 3.28 | 23.91 ± 3.19 | 0.007 | <0.001 |
| **Menopausal status, n (%)** |  |  |  |  |  |  |  |
| Postmenopausal | 1281 (44.25%) | 421 (46.37%) | 0.042 | 428 (47.19%) | 421 (46.42%) | 0.015 | <0.001 |
| **Clinical T stage, n (%)** |  |  |  |  |  |  |  |
| T1 | 408 (14.09%) | 140 (15.42%) | 0.037 | 138 (15.21%) | 139 (15.33%) | 0.003 | <0.001 |
| T2 | 1810 (62.52%) | 613 (67.51%) | 0.107 | 610 (67.25%) | 613 (67.59%) | 0.007 | <0.001 |
| T3 | 475 (16.41%) | 112 (12.33%) | 0.124 | 115 (12.68%) | 112 (12.35%) | 0.010 | <0.001 |
| T4 | 202 (6.98%) | 43 (4.74%) | 0.106 | 44 (4.85%) | 43 (4.74%) | 0.005 | <0.001 |
| **Clinical N stage, n (%)** |  |  |  |  |  |  |  |
| N0 | 1128 (38.96%) | 366 (40.31%) | 0.027 | 367 (40.46%) | 366 (40.35%) | 0.002 | <0.001 |
| N1 | 1237 (42.73%) | 384 (42.29%) | 0.009 | 391 (43.11%) | 383 (42.23%) | 0.018 | <0.001 |
| N2 | 394 (13.61%) | 109 (12.00%) | 0.049 | 100 (11.03%) | 109 (12.02%) | 0.031 | <0.001 |
| N3 | 136 (4.70%) | 49 (5.40%) | 0.031 | 49 (5.40%) | 49 (5.40%) | 0.000 | <0.001 |
| **ER status, n (%)** |  |  |  |  |  |  |  |
| Positive | 2041 (70.50%) | 429 (47.25%) | 0.466 | 423 (46.64%) | 429 (47.30%) | 0.013 | <0.001 |
| **PR status, n (%)** |  |  |  |  |  |  |  |
| Positive | 1856 (64.11%) | 371 (40.86%) | 0.473 | 387 (42.67%) | 371 (40.90%) | 0.036 | <0.001 |
| Ki-67 index, % | 41.97 ± 22.50 | 50.52 ± 21.75 | 0.393 | 50.79 ± 21.28 | 50.48 ± 21.71 | 0.014 | <0.001 |
| **IHC-defined surrogate subtype, n (%)** |  |  |  |  |  |  |  |
| Luminal A-like | 241 (8.32%) | 5 (0.55%) | 1.051 | 5 (0.55%) | 5 (0.55%) | 0.000 | <0.001 |
| Luminal B-like/HER2− | 1146 (39.59%) | 136 (14.98%) | 0.690 | 136 (14.99%) | 136 (14.99%) | 0.000 | <0.001 |
| Luminal B-like/HER2+ | 704 (24.32%) | 301 (33.15%) | 0.188 | 301 (33.19%) | 301 (33.19%) | 0.000 | <0.001 |
| ER−/HER2+ | 415 (14.34%) | 326 (35.90%) | 0.450 | 326 (35.94%) | 326 (35.94%) | 0.000 | <0.001 |
| TNBC | 389 (13.44%) | 140 (15.42%) | 0.055 | 139 (15.33%) | 139 (15.33%) | 0.000 | <0.001 |
| **Platinum-based chemotherapy, n (%)** |  |  |  |  |  |  |  |
| Yes | 500 (17.27%) | 305 (33.59%) | 0.346 | 301 (33.19%) | 304 (33.52%) | 0.007 | <0.001 |
| **Anti-HER2 therapy, n (%)** |  |  |  |  |  |  |  |
| Yes | 845 (29.19%) | 564 (62.11%) | 0.679 | 547 (60.31%) | 564 (62.18%) | 0.039 | <0.001 |
| **Calendar year of surgery, n (%)** |  |  |  |  |  |  |  |
| 2012 | 43 (1.49%) | 2 (0.22%) | 0.270 | 2 (0.22%) | 2 (0.22%) | 0.000 | <0.001 |
| 2013 | 67 (2.31%) | 8 (0.88%) | 0.153 | 6 (0.66%) | 8 (0.88%) | 0.024 | <0.001 |
| 2014 | 119 (4.11%) | 19 (2.09%) | 0.141 | 28 (3.09%) | 19 (2.09%) | 0.069 | <0.001 |
| 2015 | 125 (4.32%) | 34 (3.74%) | 0.030 | 35 (3.86%) | 34 (3.75%) | 0.006 | <0.001 |
| 2016 | 135 (4.66%) | 22 (2.42%) | 0.146 | 24 (2.65%) | 22 (2.43%) | 0.014 | <0.001 |
| 2017 | 143 (4.94%) | 21 (2.31%) | 0.175 | 27 (2.98%) | 21 (2.32%) | 0.044 | <0.001 |
| 2018 | 234 (8.08%) | 32 (3.52%) | 0.247 | 37 (4.08%) | 32 (3.53%) | 0.030 | <0.001 |
| 2019 | 625 (21.59%) | 194 (21.37%) | 0.005 | 195 (21.50%) | 194 (21.39%) | 0.003 | <0.001 |
| 2020 | 593 (20.48%) | 248 (27.31%) | 0.153 | 252 (27.78%) | 247 (27.23%) | 0.012 | <0.001 |
| 2021 | 811 (28.01%) | 328 (36.12%) | 0.169 | 301 (33.19%) | 328 (36.16%) | 0.062 | <0.001 |

Values are presented as mean ± SD for continuous variables and n (%) for categorical variables. Pathological complete response (pCR) was defined as ypT0/is ypN0. Propensity scores for achieving pCR were estimated using age, BMI, menopausal status, clinical T stage, clinical N stage, ER status, PR status, Ki-67 index, IHC-defined surrogate subtype, platinum-based chemotherapy, anti-HER2 therapy, and calendar year of surgery. Patients were matched 1:1 using nearest-neighbor matching without replacement, with exact matching on IHC-defined surrogate subtype and a caliper width of 0.2 standard deviations of the logit of the propensity score. A total of 907 of 908 patients who achieved pCR were matched to 907 of 2,895 patients without pCR; one patient with pCR and 1,988 patients without pCR were not included in the matched cohort. For overlap weighting, patients who achieved pCR were assigned weights of 1 − PS, whereas patients without pCR were assigned weights of PS. The overlap-weighted analysis retained all 3,803 observations and yielded an overall effective sample size of 2,327.2. The final column reports absolute weighted SMDs; all values were <0.001. An absolute SMD <0.10 was considered indicative of adequate covariate balance. Abbreviations: BMI, body mass index; ER, estrogen receptor; PR, progesterone receptor; HER2, human epidermal growth factor receptor 2; IHC, immunohistochemistry; OW, overlap weighting; pCR, pathological complete response; PS, propensity score; PSM, propensity score matching; SMD, standardized mean difference; TNBC, triple-negative breast cancer.

**Supplementary Table S2. Selected sensitivity analyses of the association between total pathological complete response and survival outcomes**

| **Method** | **Outcome** | **Analysis sample, n** | **Observed events, n** | **HR (95% CI)** | **P value** |
| --- | --- | --- | --- | --- | --- |
| Propensity score matching | RFS | 1814 | 77 | 0.43 (0.27–0.69) | <0.001 |
| Propensity score matching | BCSS | 1814 | 63 | 0.38 (0.22–0.64) | <0.001 |
| Propensity-score overlap weighting | RFS | 3803 | 274 | 0.38 (0.24–0.60) | <0.001 |
| Propensity-score overlap weighting | BCSS | 3803 | 239 | 0.32 (0.19–0.54) | <0.001 |
| Cox model stratified by IHC-defined surrogate subtype | RFS | 3803 | 274 | 0.37 (0.24–0.57) | <0.001 |
| Cox model stratified by IHC-defined surrogate subtype | BCSS | 3803 | 239 | 0.30 (0.18–0.50) | <0.001 |

Hazard ratios compare patients achieving pathological complete response (pCR) with non-pCR patients as the reference group; pCR was defined as ypT0/is ypN0. PSM estimates were obtained from Cox proportional hazards models with robust sandwich standard errors clustered by matched pair. Overlap-weighted estimates were obtained from weighted Cox proportional hazards models with robust standard errors to assess the association between pCR and survival in a population with overlapping propensity scores. The overlap-weighted analysis retained all 3,803 observations and yielded an overall effective sample size of 2,327.2. The same prespecified propensity-score covariates were used for PSM and overlap weighting. All prespecified covariates achieved an absolute SMD <0.10 after PSM, whereas all absolute weighted SMDs were <0.001 after overlap weighting (Supplementary Table S1). For the subtype-stratified sensitivity analysis, Cox proportional hazards models were stratified by IHC-defined surrogate subtype, allowing separate baseline hazard functions across surrogate subtypes while estimating a common association between pCR and each survival outcome. Global Schoenfeld residual tests provided no evidence of non-proportional hazards in the subtype-stratified models (RFS, P = 0.462; BCSS, P = 0.596). Abbreviations: BCSS, breast cancer-specific survival; CI, confidence interval; HR, hazard ratio; IHC, immunohistochemistry; pCR, pathological complete response; PSM, propensity score matching; RFS, recurrence-free survival; SMD, standardized mean difference.

**Supplementary Table S3. Neoadjuvant treatment regimens, calendar-year patterns of anti-HER2 therapy, and broad postoperative treatment indicators**

**A. Overall neoadjuvant treatment characteristics (N = 3,803)**

| **Variable / regimen** | **n** | **%** |
| --- | --- | --- |
| Platinum-based chemotherapy | 805 | 21.17 |
| Anti-HER2 therapy | 1,409 | 37.05 |
| **Structured neoadjuvant regimen labels** |  |  |
| EC-T | 778 | 20.46 |
| TEC | 336 | 8.84 |
| TCbH | 236 | 6.21 |
| EC-TH | 187 | 4.92 |
| EC | 125 | 3.29 |
| TCb | 70 | 1.84 |
| PCb | 41 | 1.08 |
| Other named structured regimens | 196 | 5.15 |
| Other / non-standardized free-text regimen | 1,809 | 47.57 |
| Missing | 25 | 0.66 |

**B. Anti-HER2 regimens among patients coded as receiving anti-HER2 therapy (n = 1,409)**

| **Anti-HER2 regimen** | **n** | **%** |
| --- | --- | --- |
| Trastuzumab (H) | 663 | 47.05 |
| Trastuzumab + pertuzumab (HP) | 646 | 45.85 |
| Trastuzumab + pyrotinib (HPy) | 98 | 6.96 |
| Pyrotinib (Py) | 2 | 0.14 |

**C. Anti-HER2 therapy and regimen use by calendar year of surgery**

| **Year** | **Overall cohort n** | **HER2-positive n** | **Anti-HER2 therapy n (% of HER2-positive)** | **H n (% of treated)** | **HP n (% of treated)** | **HPy n (% of treated)** | **Py n (% of treated)** |
| --- | --- | --- | --- | --- | --- | --- | --- |
| 2012 | 45 | 11 | 2 (18.2%) | 2 (100.0%) | 0 (0.0%) | 0 (0.0%) | 0 (0.0%) |
| 2013 | 75 | 25 | 15 (60.0%) | 15 (100.0%) | 0 (0.0%) | 0 (0.0%) | 0 (0.0%) |
| 2014 | 138 | 53 | 27 (50.9%) | 27 (100.0%) | 0 (0.0%) | 0 (0.0%) | 0 (0.0%) |
| 2015 | 159 | 68 | 39 (57.4%) | 39 (100.0%) | 0 (0.0%) | 0 (0.0%) | 0 (0.0%) |
| 2016 | 157 | 59 | 42 (71.2%) | 42 (100.0%) | 0 (0.0%) | 0 (0.0%) | 0 (0.0%) |
| 2017 | 164 | 70 | 46 (65.7%) | 39 (84.8%) | 7 (15.2%) | 0 (0.0%) | 0 (0.0%) |
| 2018 | 266 | 94 | 67 (71.3%) | 66 (98.5%) | 1 (1.5%) | 0 (0.0%) | 0 (0.0%) |
| 2019 | 819 | 355 | 299 (84.2%) | 243 (81.3%) | 30 (10.0%) | 26 (8.7%) | 0 (0.0%) |
| 2020 | 841 | 404 | 347 (85.9%) | 120 (34.6%) | 179 (51.6%) | 48 (13.8%) | 0 (0.0%) |
| 2021 | 1,139 | 607 | 525 (86.5%) | 70 (13.3%) | 429 (81.7%) | 24 (4.6%) | 2 (0.4%) |
| **Total** | **3,803** | **1,746** | **1,409 (80.7%)** | **663 (47.1%)** | **646 (45.8%)** | **98 (7.0%)** | **2 (0.1%)** |

**D. Broad postoperative treatment indicators by pathological complete response status**

| **Treatment indicator** | **Overall cohort (N = 3,803)** | **non-pCR (n = 2,895)** | **pCR (n = 908)** |
| --- | --- | --- | --- |
| Adjuvant chemotherapy | 969 (25.48%) | 832 (28.74%) | 137 (15.09%) |
| Radiotherapy | 2,823 (74.23%) | 2,169 (74.92%) | 654 (72.03%) |
| Endocrine therapy | 2,106 (55.38%) | 1,753 (60.55%) | 353 (38.88%) |

Values are n (%) unless otherwise indicated. Structured chemotherapy regimen codes (EC-T, TEC, TCbH, EC-TH, EC, TCb, and PCb) are reproduced exactly as recorded in the analytic database and were not reinterpreted. Because 1,809 patients (47.6%) were coded as "Other" with heterogeneous non-standardized free-text descriptions, these entries were not further collapsed into standardized regimen classes. Cycle-level information was not consistently available in the source database and therefore could not be summarized reliably. Anti-HER2 regimen distribution was summarized among patients coded as receiving anti-HER2 therapy (n = 1,409). H, trastuzumab; HP, trastuzumab plus pertuzumab; HPy, trastuzumab plus pyrotinib; Py, pyrotinib. Calendar year of surgery was used as a proxy for treatment era in sensitivity analyses. Postoperative treatment indicators were available as binary variables for all 3,803 patients. Specific regimens, treatment initiation dates, treatment duration, response-adapted escalation or de-escalation, and adherence were not available in a sufficiently standardized form. These distributions are descriptive and should not be interpreted as treatment effects because postoperative treatment occurred after pCR assessment and may have been influenced by residual disease status, tumor subtype, and other clinical factors.

**Supplementary Table S4. Association of four receptor-based clinical categories with total pathological complete response: logistic regression sensitivity analysis**

| **Clinical category** | **N** | **pCR, n (%)** | **Crude OR (95% CI)** | **Crude P** | **Adjusted OR (95% CI)** | **Adjusted P** |
| --- | --- | --- | --- | --- | --- | --- |
| HR+/HER2− | 1528 | 141 (9.23%) | 1.00 (Reference) | — | 1.00 (Reference) | — |
| HR+/HER2+ | 1098 | 342 (31.15%) | 4.45 (3.59–5.52) | <0.001 | 1.74 (1.24–2.45) | 0.001 |
| HR−/HER2+ | 648 | 285 (43.98%) | 7.72 (6.12–9.74) | <0.001 | 3.20 (2.26–4.53) | <0.001 |
| TNBC | 529 | 140 (26.47%) | 3.54 (2.73–4.59) | <0.001 | 3.45 (2.66–4.49) | <0.001 |

Values are presented as n (%) where applicable. Hormone receptor (HR) positivity was defined as estrogen receptor (ER) and/or progesterone receptor (PR) positivity, whereas HR-negative disease required both ER and PR negativity. This reclassification merged the two HER2-negative luminal categories and classified HER2-positive tumors using combined ER/PR status rather than ER status alone. Pathological complete response (pCR) was defined as ypT0/is ypN0. Odds ratios (ORs) were estimated using logistic regression, with HR-positive/HER2-negative disease as the reference category. The multivariable model was adjusted for age, body mass index, clinical T stage, clinical N stage, platinum-based chemotherapy, and anti-HER2 therapy. Abbreviations: CI, confidence interval; ER, estrogen receptor; HER2, human epidermal growth factor receptor 2; HR, hormone receptor; OR, odds ratio; pCR, pathological complete response; PR, progesterone receptor; TNBC, triple-negative breast cancer.

**Supplementary Table S5. Data completeness for variables reported or used in the study analyses**

| **Variable** | **Role in manuscript/analysis** | **Available, n (%)** | **Missing, n (%)** |
| --- | --- | --- | --- |
| Age | Primary covariate | 3,803 (100.00%) | 0 (0.00%) |
| Body mass index (BMI) | Primary covariate | 3,803 (100.00%) | 0 (0.00%) |
| Menopausal status | PS sensitivity covariate | 3,803 (100.00%) | 0 (0.00%) |
| Clinical T stage | Primary covariate | 3,803 (100.00%) | 0 (0.00%) |
| Clinical N stage | Primary covariate | 3,803 (100.00%) | 0 (0.00%) |
| ER status | Subtype component / PS covariate | 3,803 (100.00%) | 0 (0.00%) |
| PR status | Subtype component / PS covariate | 3,803 (100.00%) | 0 (0.00%) |
| HER2 status | Subtype component | 3,803 (100.00%) | 0 (0.00%) |
| Ki-67 index | Subtype component / PS covariate | 3,803 (100.00%) | 0 (0.00%) |
| IHC-defined surrogate subtype | Primary covariate | 3,803 (100.00%) | 0 (0.00%) |
| Calendar year of surgery | Treatment-era sensitivity covariate | 3,803 (100.00%) | 0 (0.00%) |
| Platinum-based chemotherapy | Primary treatment covariate | 3,803 (100.00%) | 0 (0.00%) |
| Anti-HER2 therapy | Primary treatment covariate | 3,803 (100.00%) | 0 (0.00%) |
| Anti-HER2 regimen among treated patients | Descriptive treatment variable | 1,409 (100.00%) | 0 (0.00%) |
| Structured neoadjuvant regimen label | Descriptive treatment variable | 3,778 (99.34%) | 25 (0.66%) |
| Breast pCR | Secondary pathological-response variable | 3,803 (100.00%) | 0 (0.00%) |
| Total pCR | Primary outcome | 3,803 (100.00%) | 0 (0.00%) |
| Adjuvant chemotherapy | Descriptive postoperative treatment variable | 3,803 (100.00%) | 0 (0.00%) |
| Radiotherapy | Descriptive postoperative treatment variable | 3,803 (100.00%) | 0 (0.00%) |
| Endocrine therapy | Descriptive postoperative treatment variable | 3,803 (100.00%) | 0 (0.00%) |
| RFS follow-up time | Survival endpoint component | 3,803 (100.00%) | 0 (0.00%) |
| RFS event indicator | Survival endpoint component | 3,803 (100.00%) | 0 (0.00%) |
| BCSS follow-up time | Survival endpoint component | 3,803 (100.00%) | 0 (0.00%) |
| BCSS event indicator | Survival endpoint component | 3,803 (100.00%) | 0 (0.00%) |

Notes: This table includes variables reported in the manuscript or used in the primary and sensitivity analyses. Percentages are calculated using the full analytic cohort (N = 3,803), except for anti-HER2 regimen, for which the denominator is the 1,409 patients coded as receiving anti-HER2 therapy. All variables required for the primary multivariable logistic and Cox regression models were complete, and no imputation was performed. The structured neoadjuvant regimen label was unavailable for 25 patients (0.66%); regimen-level information was therefore summarized descriptively and was not imputed. Conditional free-text treatment fields are not shown because blank entries may indicate non-applicability rather than missingness. Cycle-level neoadjuvant treatment information was not consistently available, and patient-level center identifiers were not available in the analytic extract; these features therefore could not be summarized as conventional item-level missing-data variables. Broad indicators of adjuvant chemotherapy, radiotherapy, and endocrine therapy were complete for all 3,803 patients; however, specific regimens, treatment initiation dates, treatment duration, modifications, and adherence were not available in a sufficiently standardized form.

Abbreviations: BCSS, breast cancer-specific survival; BMI, body mass index; ER, estrogen receptor; HER2, human epidermal growth factor receptor 2; pCR, pathological complete response; PR, progesterone receptor; PS, propensity score; RFS, recurrence-free survival.

**Supplementary Table S6. Survival event counts by IHC-defined surrogate subtype and total pathological complete response status, and diagnostics for subtype-specific Cox models**

**Panel A. Event counts by subtype and pCR status**

| **Subtype** | **pCR status** | **N** | **RFS events** | **BCSS deaths** |
| --- | --- | --- | --- | --- |
| Luminal A-like | non-pCR | 241 | 7 | 7 |
| Luminal A-like | pCR | 5 | 0 | 0 |
| Luminal B-like/HER2− | non-pCR | 1,146 | 108 | 94 |
| Luminal B-like/HER2− | pCR | 136 | 6 | 3 |
| Luminal B-like/HER2+ | non-pCR | 704 | 36 | 32 |
| Luminal B-like/HER2+ | pCR | 301 | 4 | 2 |
| ER−/HER2+ | non-pCR | 415 | 39 | 35 |
| ER−/HER2+ | pCR | 326 | 8 | 8 |
| TNBC | non-pCR | 389 | 61 | 54 |
| TNBC | pCR | 140 | 5 | 4 |

**Panel B. Diagnostics for subtype-specific Cox models**

| **Subtype** | **Outcome** | **N** | **Events** | **Specified coefficients** | **Fitted parameters** | **Non-estimable/aliased terms** | **Diagnostic** |
| --- | --- | --- | --- | --- | --- | --- | --- |
| Luminal A-like | RFS | 246 | 7 | 11 | 9 | Platinum-based chemotherapy; Anti-HER2 therapy | Monotone-likelihood warning; pCR HR not estimable |
| Luminal A-like | BCSS | 246 | 7 | 11 | 9 | Platinum-based chemotherapy; Anti-HER2 therapy | Monotone-likelihood warning; pCR HR not estimable |
| Luminal B-like/HER2− | RFS | 1,282 | 114 | 11 | 10 | Anti-HER2 therapy | No warning; finite coefficients and standard errors |
| Luminal B-like/HER2− | BCSS | 1,282 | 97 | 11 | 10 | Anti-HER2 therapy | No warning; finite coefficients and standard errors |
| Luminal B-like/HER2+ | RFS | 1,005 | 40 | 11 | 11 | None | No warning; finite coefficients and standard errors |
| Luminal B-like/HER2+ | BCSS | 1,005 | 34 | 11 | 11 | None | No warning; finite coefficients and standard errors |
| ER−/HER2+ | RFS | 741 | 47 | 11 | 11 | None | No warning; finite coefficients and standard errors |
| ER−/HER2+ | BCSS | 741 | 43 | 11 | 11 | None | No warning; finite coefficients and standard errors |
| TNBC | RFS | 529 | 66 | 11 | 10 | Anti-HER2 therapy | No warning; finite coefficients and standard errors |
| TNBC | BCSS | 529 | 58 | 11 | 10 | Anti-HER2 therapy | No warning; finite coefficients and standard errors |

Note 1. The specified model included 11 regression coefficients: pCR, age, BMI, three clinical T-stage coefficients, three clinical N-stage coefficients, platinum-based chemotherapy, and anti-HER2 therapy.

Note 2. The baseline hazard was not counted as a fitted parameter. The fitted-parameter count equals the number of non-NA regression coefficients (the log-likelihood degrees of freedom).

Note 3. Terms with non-estimable coefficients (NA) were not counted as fitted parameters; this may reflect absent within-subtype variation or insufficient information in the event risk sets.

Note 4. Absence of a numerical convergence warning does not establish estimate stability. Because of sparse events, all subtype-specific estimates should be interpreted as exploratory.

Abbreviations: BCSS, breast cancer-specific survival; BMI, body mass index; HER2, human epidermal growth factor receptor 2; HR, hazard ratio; IHC, immunohistochemistry; NA, not available/not estimable; pCR, pathological complete response; RFS, recurrence-free survival; TNBC, triple-negative breast cancer.

**Supplementary Table S7. Proportional hazards diagnostics and subtype-stratified sensitivity analyses for the four-subtype pCR-by-subtype interaction models**

**Panel A. Schoenfeld residual tests for the conventional four-subtype interaction models**

| **Outcome** | **N** | **Events** | **Model term** | **Schoenfeld χ²** | **df** | **P value** |
| --- | --- | --- | --- | --- | --- | --- |
| RFS | 3,557 | 267 | pCR | 2.124 | 1 | 0.145 |
| RFS | 3,557 | 267 | IHC-defined surrogate subtype | 3.545 | 3 | 0.315 |
| RFS | 3,557 | 267 | Age | 0.972 | 1 | 0.324 |
| RFS | 3,557 | 267 | BMI | 2.532 | 1 | 0.112 |
| RFS | 3,557 | 267 | Clinical T stage | 2.455 | 3 | 0.484 |
| RFS | 3,557 | 267 | Clinical N stage | 0.646 | 3 | 0.886 |
| RFS | 3,557 | 267 | Platinum-based chemotherapy | 0.010 | 1 | 0.921 |
| RFS | 3,557 | 267 | Anti-HER2 therapy | 1.410 | 1 | 0.235 |
| RFS | 3,557 | 267 | pCR × subtype interaction | 2.662 | 3 | 0.447 |
| RFS | 3,557 | 267 | Global test | 15.677 | 17 | 0.547 |
| BCSS | 3,557 | 232 | pCR | 1.197 | 1 | 0.274 |
| BCSS | 3,557 | 232 | IHC-defined surrogate subtype | 3.833 | 3 | 0.280 |
| BCSS | 3,557 | 232 | Age | 0.103 | 1 | 0.749 |
| BCSS | 3,557 | 232 | BMI | 2.428 | 1 | 0.119 |
| BCSS | 3,557 | 232 | Clinical T stage | 2.262 | 3 | 0.520 |
| BCSS | 3,557 | 232 | Clinical N stage | 1.528 | 3 | 0.676 |
| BCSS | 3,557 | 232 | Platinum-based chemotherapy | 0.116 | 1 | 0.733 |
| BCSS | 3,557 | 232 | Anti-HER2 therapy | 1.589 | 1 | 0.208 |
| BCSS | 3,557 | 232 | pCR × subtype interaction | 1.776 | 3 | 0.620 |
| BCSS | 3,557 | 232 | Global test | 13.597 | 17 | 0.695 |

**Panel B. Likelihood-ratio tests of the pCR-by-subtype interaction under alternative baseline-hazard specifications**

| **Outcome** | **Baseline-hazard specification** | **N** | **Events** | **Reduced-model parameters** | **Full-model parameters** | **Likelihood-ratio χ²** | **df** | **P value** |
| --- | --- | --- | --- | --- | --- | --- | --- | --- |
| RFS | Conventional Cox model (subtype entered as a main effect) | 3,557 | 267 | 14 | 17 | 1.576 | 3 | 0.665 |
| RFS | Cox model stratified by subtype (separate baseline hazards) | 3,557 | 267 | 11 | 14 | 1.831 | 3 | 0.608 |
| BCSS | Conventional Cox model (subtype entered as a main effect) | 3,557 | 232 | 14 | 17 | 1.349 | 3 | 0.718 |
| BCSS | Cox model stratified by subtype (separate baseline hazards) | 3,557 | 232 | 11 | 14 | 1.720 | 3 | 0.633 |

Note 1. Analyses used the original unmatched and unweighted cohort and were restricted to four IHC-defined surrogate subtypes with estimable within-subtype pCR contrasts: Luminal B-like/HER2−, Luminal B-like/HER2+, ER−/HER2+, and TNBC. Luminal A-like disease was excluded because none of the five patients who achieved pCR experienced an RFS event or breast cancer-specific death.

Note 2. All Cox models adjusted for age, BMI, clinical T stage, clinical N stage, platinum-based chemotherapy, and anti-HER2 therapy; tied event times were handled using the Efron approximation.

Note 3. Panel A reports term-specific and global tests based on scaled Schoenfeld residuals from the full conventional interaction model, using the Kaplan–Meier transformation. Multi-parameter factor and interaction terms were tested jointly. Under treatment coding, the standalone pCR row pertains to the Luminal B-like/HER2− reference subtype and is not an omnibus pCR test across all four subtypes.

Note 4. In Panel B, the conventional reduced model contained pCR and subtype main effects and the full model additionally contained the three pCR-by-subtype interaction terms. For the subtype-stratified analysis, the reduced model estimated one common pCR coefficient and the full model estimated four subtype-specific pCR coefficients; the resulting likelihood-ratio test had 3 degrees of freedom.

Note 5. Baseline hazard functions were not counted as fitted regression parameters. All eight reduced and full Cox models converged without warnings, and all fitted coefficients and standard errors were finite. Nonsignificant Schoenfeld tests indicate no statistical evidence of non-proportional hazards but do not prove proportionality.

Abbreviations: BCSS, breast cancer-specific survival; BMI, body mass index; df, degrees of freedom; ER, estrogen receptor; HER2, human epidermal growth factor receptor 2; IHC, immunohistochemistry; LRT, likelihood-ratio test; pCR, pathological complete response; RFS, recurrence-free survival; TNBC, triple-negative breast cancer.

**Supplementary Figure S1. Covariate balance before and after propensity score matching.** Absolute SMDs are shown for the prespecified covariates. Propensity scores for achieving total pCR were estimated using age, body mass index, menopausal status, clinical T stage, clinical N stage, estrogen receptor status, progesterone receptor status, continuous Ki-67 index, IHC-defined surrogate subtype, platinum-based chemotherapy, anti-HER2 therapy, and calendar year of surgery. Patients were matched 1:1 using nearest-neighbor matching without replacement, with exact matching on IHC-defined surrogate subtype and a caliper of 0.2 standard deviations of the logit of the propensity score, yielding 907 matched pairs. The dashed vertical line at 0.10 denotes the threshold for adequate covariate balance. Total pCR was defined as ypT0/is ypN0. Abbreviations: ER, estrogen receptor; HER2, human epidermal growth factor receptor 2; IHC, immunohistochemistry; pCR, pathological complete response; PR, progesterone receptor; SMD, standardized mean difference; TNBC, triple-negative breast cancer.

**Supplementary Figure S2.** **Flow diagram of the available analytic cohort and survival analyses.** The available analytic cohort comprised 3,803 patients who met the prespecified eligibility criteria: 908 achieved total pCR, defined as ypT0/is ypN0, and 2,895 did not achieve pCR. All 3,803 patients were included in the survival analyses, with 274 RFS events and 239 breast cancer-specific deaths. Source-level screening and criterion-specific exclusion counts preceding construction of the available analytic extract were not retained and could not be reliably reconstructed. Abbreviations: BCSS, breast cancer-specific survival; pCR, pathological complete response; RFS, recurrence-free survival; SJTU-BCDB, Shanghai Jiao Tong University Breast Cancer Database.
